# Could shared decision making affect staying in hospital? A cross-sectional pilot study

**DOI:** 10.1186/s12913-019-4002-8

**Published:** 2019-03-18

**Authors:** M. R. Gualano, F. Bert, S. Passi, M. Stillo, V. Brescia, G. Scaioli, R. Thomas, G. Voglino, D. Minniti, F. Boraso, R. Siliquini

**Affiliations:** 1 Department of Public Health Sciences, University of Turin, Via Santena 5 bis, 10126 Turin, Italy; 2 AOU Città della Salute e della Scienza, Torino, Italy; 3 Local Health Unit, ASL TO 3, Piedmont, Italy; 4 Department of Management, University of Torino, Torino, Italy

**Keywords:** Share decision making, Length of stay, Health expenditure, Patient-doctor relationship

## Abstract

**Background:**

Shared Decision Making (SDM) is an approach where clinicians and patients share the best available evidence to make decision and where patients opinions are considered. This approach provides benefits for patients, clinicians and health care system. The aim of the present study is to investigate the patients’ perception of their participation in treatment choices and to identify the possible influences of variables in decision aids and therapeutic choices. Furthermore the present study evaluates the impact of SDM on the length of hospital stay and the health expenditure in Piemonte, an Italian region.

**Methods:**

A cross-sectional study was performed in 2016. The patients were selected after hospitalization to clinical and surgical units at the Rivoli and Susa Hospital. Data were collected through the questionnaire and the Hospital Discharge Registers. STROBE guidelines for observational studies were used. A descriptive analysis was conducted. Frequencies and percentages of the categorical variables were reported. Statistical analyses were performed using t-test, chi-square test and Mann-Whitney test.

**Results:**

The final sample was made of 174 subjects. More than half of the sample reported a SDM approach. Female gender (*p* = 0.027) and lower age (*p* = 0.047) are associated with an increased possibility to report SDM. Receiving “good” or “excellent” information, having their own request fulfilled and their opinions took into account by healthcare professionals, were all found to be predictors for an approach recognized as SDM (*p* ≤ 0.05). The perception that healthcare professionals spent a proper amount of time with the patients and used an understendable language are factors increase the chance of a “shared” decision process (*p* ≤ 0.05). The patients trust in the information given by the healthcare professional is not affecting their perception about the decision making process (*P* = 0.195). No significant difference where recorded in length of stay and hospital expenditure.

**Conclusions:**

The data show the role played by different dimension of the patients-clinician relationship and that the strongest determinant of a perceived shared decision making approach are healthcare professional-depending.

## Background

Shared decision making (SDM) has been defined as: “an approach where clinicians and patients share the best available evidence when faced with the task of making decisions, and where patients are supported to consider options, to achieve informed preferences” [1].

In medicine three different models of patient-doctor relationship can be recognized. The *“Paternalistic Model”*, where the patient passively acquiesces to professional authority by agreeing to the doctor’s choice of treatment, the *“Informed Model”* in which the doctor communicates to the patient information on treatment options, risks and benefits in order to enable the patient to take an informed treatment decision and, lastly, the *“Shared model”* [2, 3]. In the *“Shared Model”*, doctors and patients simultaneously share all the stages of the decisions related to the treatment process, recognizing the need to support autonomy by building good relationships, respecting both individual competence and interdependence on others [4, 5].

### Shared decision making main features

The earliest mention of SDM was made in 1982 but the idea comes from and deepens through the principles of patient centered care [6, 7]. Elwyn et al. in 2012 proposed a method to apply SDM in routing settings. According to this paper, in fact, achieving shared decision making depends on building a good relationship in the clinical encounter, so that the information is shared and patients are supported to deliberate. This model rests on supporting a process of deliberation, and on understanding that decisions should be influenced by exploring and respecting “what matters most” to individuals. [8] Furthermore, different studies showed how SDM has the potential to provide numerous benefits for patients, clinicians, and the health care system. In particular, these benefits included an increase in patients’ knowledge and satisfaction. Furthermore SDM seems to reduce anxiety, improve outcomes and reduce health costs [9–13].

### Shared decision making diffusion and prospective

However, for many decades and still nowadays, the dominant approach in decision making about treatment in the medical encounter has been represented by the “*Paternalistic Model”* [13] and some health care professional still express their doubts about the *“Shared Decision Model”*, arguing that patients do not really want to get involved in decision making, they are not qualified to do it, thus they might take “bad” decisions. In other scenarios, some healthcare professionals claim they are already using the SDM, but data from patient experience surveys indicates that this effort is not perceived by the patients. [14, 15]. In addition, a majority of patients do express a desire to have a role in SDM, emphasizing the need for furtherly developed evidence on how to facilitate such process [16–21].

Nowadays, numerous efforts are being implemented in public health to find a way to guarantee the best health care for the population, searching for the highest efficacy and efficiency despite the resources reduction; one of the implemented measures could be to avoid inappropriate interventions and to reduce inadequate and long hospitalization periods. The call for doctors-patients partnership opens up options beyond paternalism, in order to approach the task of making decisions about treatment and it is part of a broader context that includes business case and costs of health [22–24]. Currently, few studies described the real economic impact of SDM with discordant results [25–28].

### Aims of the study

The aim of the present cross-sectional pilot study is to investigate how patients perceive the opportunity of participation in treatment choices, and to identify the socio-demographic and patient-doctor relationship features that may influence decision aids and therapeutic choices.

Secondly, the study wants to evaluate if and how the length of hospital stay and the health expenditure in a hospital setting are influenced by the implementation of SDM instead of other decision making models.

## Methods

In order to achieve the main purpose of the present study we performed a cross-sectional pilot study between January and April 2016. STROBE guidelines for observational studies were used for reporting [29].

### The sample

Participation was voluntary, anonymous and without compensation. The study was approved by the Internal Review Board of the Department of Public Health Sciences of the University of Torino, Italy. The interviewers ensured anonymity of participants and the maintenance of ethical principles. The patients were selected after hospitalization to clinical and surgical units at the Rivoli and Susa Hospital (Health Care Units Turin 3 ASLTO3, Piedmont, Italy). All patients hospitalized for at least one day were included in the study. Those patients who could not make decisions about their health, because minors or unable, were excluded. Furthermore, underage subjects and those who were not able to understand the questionnaire were excluded. In particular, prior to the administration of the questionnaire, the background and the objectives of the study were explained, and subjects were asked to sign an informed consent form.

All the patients that were hospitalized during the study period were asked to participate if they were eligible. In order to define the sample size we considered the mean number of hospitalization of patients with the inclusion criteria in the previous year (*N* = 240), a confidence level of 95% and a distribution (that reflects how skewed the respondents are on a topic) of 50%, which we considered as the most conservative.

The sample size was then calculated using the following formula:

Sample Size = (Distribution of 50%) / (Margin of Error% / Confidence Level Score)2.

In order to identify the true sample, the result of this calculation was then corrected according to the following formula:

True Sample = (Sample Size x Population) / (Sample Size + Population – 1), where population, as previously reported, was represented by the mean number of hospitalization in the previous year (N = 240). The final sample was determined as 148 interviews.

### Data collection

Data were collected through a paper based, non-self-compiling structured questionnaire and the Hospital Discharge Registers. Two trained resident doctors performed the interviews on fixed days, considering the average length of stay in each single ward involved and they performed direct personal interviews using a twenty-six items structured questionnaire. The questionnaire was developed after a review of studies on this topic in scientific databases [1, 4, 10, 18]. The interview lasted approximately 10 min. The questionnaire assessed: socio-demographic, health status and economic variables. As well as dichotomous variables, other subjective variables were assessed through a Likert scale system: health status, quality of medical information and languages, trust in the healthcare system and doctors, and medical skills. The participation in treatment choices was derived using a dichotomous variable. The length of hospital stay and the data regarding the health expenditure were extracted, for each patient involved, from the Hospital Discharge Register and the Diagnosis Related Group (DRG). The length of hospital stay was measured as median duration of hospital stay (in days) and the health expenditure was expressed in Euro. These data were asked to the ASL TO3 Health Directorate.

### Statistical analysis

Statistical analyses were conducted with STATA MP13 software (Stata Corp., College Station, TX, 2013). A descriptive analysis of the sample was conducted and results were expressed in frequencies and percentages for categorical variables or through mean and standard deviation for continuous variables. Statistical analyses were performed using t-test, chi-square test, Fisher’s exact test and Mann Whitney test. Significance level was set at *p* = 0.05.

## Results

The final sample was made of 174 subjects. The mean age was 66.8 ± 17.8 years old, with the youngest inpatients aged 20 and the eldest 94. The average length of stay resulted to be 13.1 ± 10.3 days and the average expenditure for hospitalization was 2709.4 ± 1542.7 €, ranging from 728 € to 9174 €.

The socio-economic features of the sample and the results obtained through chi-square tests are reported in Table 1. These tests were performed to investigate whether the independent variables related to socio-demographic characteristics, health status and economic situation played a role in influencing patients’ attitude and opinion about various aspects of the received treatment process, healthcare operators/patients relationship and about the opportunity of an informed and shared decision process.

**Table 1:** Socio economic characteristics stratified by the rating of the relationship with healthcare professionals

		How would you rate your relationship with healthcare professionals?					*P*-value
		Poor N (%)	Fair N (%)	Good N (%)	Excellent N (%)	Total	
Gender	Male	2 (2.5)	23 (28.1)	32 (39.8)	24 (29.6)	81	0.007
	Female	1 (1.1)	10 (11.1)	57 (63.3)	22 (24.5)	90	
Age	< 40 years old	1 (7.1)	0 (0)	10 (71.4)	3 (21.5)	14	0.087
	Between 40 and 60 years old	0 (0)	5 (15.2)	22 (66.7)	6 (8.1)	33	
	> 60 years old	2 (1.6)	27 (22)	57 (46.3)	37 (30.1)	123	
Marital Status	Unmarried	0 (0)	4 (16.7)	15 (62.5)	5 (20.8)	24	0.828
	Cohabitant	0 (0)	1 (9.1)	8 (72.7)	2 (18.2)	11	
	Married	3 (3.4)	20 (23.1)	39 (44.8)	25 (28.7)	87	
	Widow	0 (0)	7 (18.9)	20 (54.1)	10 (27)	37	
	Divorced	0 (0)	2 (15.4)	7 (53.8)	4 (30.8)	13	
Educational Status	None	0 (0)	0 (0)	7 (87.5)	1 (12.5)	8	0.929
	Primary School	1 (2)	10 (20)	24 (48)	15 (30)	50	
	Middle School	1 (2)	11 (22.4)	25 (51)	12 (24.5)	49	
	High School	1 (1.8)	12 (21.4)	29 (51.8)	14 (25)	56	
	Graduation	0 (0)	1 (11.1)	5 (55.6)	3 (33.3)	9	
Previous hospitalization for the same reason	Yes	0 (0)	17 (31.5)	24 (44.4)	13 (24.1)	54	0.074
	No	1 (1)	15 (14.6)	61 (59.2)	26 (25.2)	103	
Hospitalization in the last 3 years for other reason	Yes	1 (1.8)	10 (17.9)	27 (48.2)	18 (32.1)	56	0.664
	No	2 (1.8)	24 (21.4)	60 (53.6)	26 (23.2)	112	

The first analyzed outcome, as presented in Table 1, consisted of a rating of the relationship with healthcare professionals; the only significant difference inside the sample group (*p* = 0.007) was found for the gender variable. While 23 (28.1%) male patients rated it as “fair”, just 10 females (11.1%) shared this opinion too. On the other hand, 32 males (39.8%) rated it as good, whereas 57 females (63.3%) provided the same rating. The second question was “How would you rate the information you received about your health status?”. In this case, as reported in Table 2, the only significant difference (*p* = 0.026) noticed in the patients’ answers was between those who had been previously hospitalized for the same reason and those who had not been. Particularly, the previously hospitalized patients received information was “fair” for 12 patients (21.8%), “good” for 19 (34.5%) and “excellent” for 21 (38.2%), while for not previously hospitalized it was “fair” in 9 cases (8.7%), “good” in 59 (57.3%) and “excellent” in 31 (30.1%).

**Table 2:** Socio economic characteristics of the sample stratified by the rating of the information received

		How would you rate the information you received about your health status?					*P*-value
		Poor N (%)	Fair N (%)	Good N (%)	Excellent N (%)	Total	
Gender	Male	5 (6.2)	11 (13.6)	40 (49.4)	25 (30.8)	81	0.610
	Female	2 (2.2)	14 (15.6)	44 (48.9)	30 (33.3)	90	
Age	< 40 years old	0 (0)	2 (14.3)	8 (57.1)	4 (28.6)	14	0.831
	Between 40 and 60 years old	1 (3)	3 (9.1)	15 (45.5)	14 (42.4)	33	
	> 60 years old	5 (4.1)	20 (16.3)	59 (47.9)	39 (31.7)	123	
Marital Status	Unmarried	2 (8.3)	3 (12.5)	13 (54.2)	6 (25)	24	0.413
	Cohabitant	0 (0)	1 (9)	5 (45.5)	5 (45.5)	11	
	Married	4 (4.5)	14 (15.9)	43 (48.9)	28 (30.7)	88	
	Widow	2 (5.4)	6 (16.2)	20 (54.1)	9 (24.3)	37	
	Divorced	0 (0)	1 (7.7)	3 (23.1)	9 (69.2)	13	
Educational Status	None	0 (0)	1 (12.5)	3 (37.5)	4 (50)	8	0.327
	Primary School	3 (5.9)	11 (21.6)	24 (47)	13 (25.5)	51	
	Middle School	4 (8.3)	6 (12.5)	20 (41.7)	18 (37.5)	48	
	High School	0 (0)	5 (8.9)	33 (58.9)	18 (32.2)	56	
	Graduation	0 (0)	2 (22.2)	3 (33.3)	4 (44.5)	9	
Previous hospitalization for the same reason	Yes	3 (5.5)	12 (21.8)	19 (34.5)	21 (38.2)	55	0.026
	No	4 (3.9)	9 (8.7)	59 (57.3)	31 (30.1)	103	
Hospitalization in the last 3 years for other reason	Yes	1 (1.8)	9 (15.8)	29 (50.9)	18 (31.5)	57	0.659
	No	6 (5.4)	14 (12.5)	54 (48.2)	38 (33.9)	112	

The questionnaire also assessed if the interviewed patients felt their diagnostic/therapeutic process during hospitalization as a process of “Shared Decision Making”. This variable was considered as binary. As reported in Table 3, more than half of males patients (*N* = 44; 53.7%) reported their experience as a shared decision process, versus 63 (70%) of the female patients; this difference was proved to be statistically significant (*p* = 0.027). For the same item, 10 out of 14 (71.4%) patients younger than 40 years old claimed there was a shared decision, for those between 40 and 60 years old (33 total) the same answer was given by 26 (78.8%) patients, while these percentages fall to 56.5% (*N* = 70 out of 124) for patients older than 60 years.

**Table 3:** Socio economic characteristics of the sample stratified by perceived shared decision making

		Shared Decision Making?			
		No N (%)	Yes N (%)	Total	P-value
Gender	Male	38 (46.3)	44 (53.7)	82	0.027
	Female	27 (30)	63 (70)	90	
Age	< 40 years old	4 (28.6)	10 (71.4)	14	0.047
	Between 40 and 60 years old	7 (21.2)	26 (78.8)	33	
	> 60 years old	54 (43.5)	70 (56.5)	124	
Marital Status	Unmarried	7 (29.2)	17 (70.8)	24	0.641
	Cohabitant	6 (54.5)	5 (45.4)	11	
	Married	35 (39.8)	53 (60.2)	88	
	Widow	15 (40.5)	22 (59.5)	37	
	Divorced	4 (30.8)	9 (69.2)	13	
Educational Status	None	3 (37.5)	5 (62.5)	8	0.731
	Primary School	23 (45.1)	28 (54.9)	51	
	Middle School	19 (32.2)	30 (50.8)	59	
	High School	18 (32.1)	38 (67.9)	56	
	Graduation	4 (44.4)	5 (55.6)	9	
Previous hospitalization for the same reason	Yes	23 (41.8)	32 (58.2)	55	0.545
	No	38 (36.9)	65 (63.1)	103	
Hospitalization in the last 3 years for other reason	Yes	25 (43.9)	32 (56.1)	57	0.303
	No	40 (35.7)	72 (64.3)	112	

After performing the Mann-Whitney test to compare the length of stay between the population who declared a SDM approach and who did not, no significant differences were noted (*p* = 0.123). Moreover, the same test was also conducted considering hospital expenditure, and neither this time it returned a significant difference between the two populations assessed (*p* = 0.08).

Finally, the researchers tested through a chi-square analysis whether the probability of reporting a shared decision diagnostic/therapeutic process by the patients may be associated with patients’ attitude and opinion about various aspects of the received information during their hospitalization, the perceived quality of healthcare operators/patients relationship, the clarity of information, availability of the operators and the trust in doctors’ decisions. This data is reported in Table 4. Referring to received information, all patients who reported “poor” in the questionnaire (*N* = 7; 100%) did not feel their hospitalization as managed with shared decisions. On the other hand, the majority of those who thought they received “good” (*N* = 53; 63.1%) and especially “excellent” (*N* = 42; 73.7%) information felt like their treatments decisions were shared between them and the doctors. All results were statically significant (*p* < 0.001). It is also possible to notice how the feeling of a shared decision process is lower when the reported frequency of patients’ requests fulfillment was “never” (*N* = 2; 33.3%) and “rarely” (*N* = 21; 46.7%) in respect of when it was “very often” (*N* = 30; 71.4%) and “always” (*N* = 50; 71.4%). All results were statically significant (*p* = 0.008).

**Table 4:** Results from the Likert evaluation stratified by perceived shared decision making

		Shared Decision Makking?			
		No N (%)	Yes N (%)	Total	P-value
How would you rate the information you received about your health status?	Poor	7 (100)	0 (0)	7	< 0.001
	Fair	14 (56)	11 (44)	25	
	Good	31 (36.9)	53 (63.1)	84	
	Excellent	15 (26.3)	42 (73.7)	57	
How many times have your requests been fulfilled by healthcare professionals?	Never	4 (66.7)	2 (33.3)	6	0.008
	Rarely	24 (53.3)	21 (46.7)	45	
	Very Often	12 (28.6)	30 (71.4)	42	
	Always	20 (28.6)	50 (71.4)	70	
	I don’t know	6 (66.7)	3 (33.3)	9	
During your hospitalization how often the healthcare professionals used a language easy to understand?	Never	4 (80)	1 (20)	5	< 0.001
	Rarely	26 (70.3)	11 (29.7)	37	
	Very Often	17 (34.7)	32 (65.3)	49	
	Always	17 (21.5)	62 (78.5)	79	
	I don’t know	3 (100)	0 (0)	3	
During your hospitalization how often you had the feeling that your opinions were took in account by healthcare professionals?	Never	13 (76.5)	4 (23.5)	17	< 0.001
	Rarely	19 (55.9)	15 (44.1)	34	
	Very Often	10 (21.3)	37 (78.7)	47	
	Always	13 (24.1)	41 (75.9)	54	
	I don’t know	11 (55)	9 (45)	20	
During your hospitalization how often did you have the feeling that healthcare professionals were spending a proper amount of time taking care of you?	Never	8 (80)	2 (20)	10	0.005
	Rarely	19 (45.2)	23 (54.8)	42	
	Very Often	20 (35.1)	37 (64.9)	57	
	Always	15 (25.4)	44 (74.6)	59	
	I don’t know	3 (75)	1 (25)	4	
How much do you trust the information you received from healthcare professionals?	Not at all	1 (100)	0 (0)	1	0.195
	Moderately	10 (55.6)	8 (44.4)	18	
	Very	34 (37.4)	57 (62.6)	91	
	Extremely	20 (32.8)	41 (67.2)	61	

Furthermore, most of the patients who thought that healthcare professionals “never” or “rarely” used a language easy to understand also reported no shared decision making (N = 4, 80%; *N* = 26, 70.3% respectively; *p* < 0.001). On the other hand, high percentages of shared decision making were associated to those patients who claimed that the language was “very often” or “always” easy to interpret (*N* = 32, 65.3%; *N* = 62, 78.5% respectively; p < 0.001). Regarding the perceived availability of the healthcare professionals to take into account patients’ opinions, most patients who claimed it was “never” (*N* = 13, 76.5%; p < 0.001) also felt the absence of shared decisions, while the large majority of those who felt it was “very often” or “always” (*N* = 37, 78.7%; *N* = 41, 75.9% respectively; p < 0.001) felt, at the same time, that they were receiving a shared decision treatment.

Lastly, 80% of the interviewed patients (*N* = 8) who “never” felt that healthcare professionals were spending the right amount of time on their care also felt that decisions were not shared between them and the doctors. This contrasts with the numerous patients who felt that “very often” and “always” the amount of time spent on their healthcare was appropriated and that, at the same time, felt that decisions upon their hospitalization were shared (N = 37, 64.9%; *N* = 44, 74.6% respectively; *p* < 0.005). Nevertheless, no statistically significant results were found for the different feelings about patients’ trust towards information received from the healthcare professionals.

## Discussion

Shared decision making represents a model of patient-doctor relationship connected to the patient centered care. The impact of this type of approach has been evaluated in different studies worldwide, all assessing different settings and conditions [30–34]. Systematic reviews underline how this type of relationship could not only improve drug adherence and therapy compliance, disease control and a reduction of the number of healthcare visits [30, 33], but it could also improve the quality of life, patients’ satisfaction and lower decisional conflict [31, 33].

On the other hand, there is controversy about the successful application of shared decision-making tools, such as time constraints or utility in lower literacy populations and different cultures [35, 36]. Furthermore, there is weak evidence that supports shared decision-making. The existing literature largely assessed and mainly focused upon patient involvement, thus only capturing one side of the shared decision-making construct, resulting in poor quality outcomes, which still need further robust studies examining all aspects of the association [34].

### Effects on hospitalization

In our study more than the half of the interviewed subjects declared that they had received a shared decision making approach. Although, based on study results’, this type of approach did not provide a significant reduction on the length of hospitalization nor on the expenditure for hospitalization of the patients involved by their doctors into a shared decision approach.

### Relations with socio-demographic and patient-doctor relationship features

Interestingly, some socio-economic features, like female gender and younger age (less than 60 years old), appeared to be positive predictors for a SDM approach, while, taking into account the patient-doctor relationship features, receiving “good” or “excellent” information, having their own request fulfilled and their opinions took into account by healthcare professionals, were also found to be predictors for an approach recognized as SDM by the patients.

Moreover, the perception that healthcare professionals spent a proper amount of time with the patients and used a language easy to understand are other factors affecting the possibility to consider the decision process as “shared”. On the other hand, the patients trust in the information given by the healthcare professional is not affecting their perception about the decision making process, thus demonstrating how this is still a key-feature involved in all decision-making processes.

Overall, these data point out how the strongest determinants of a perceived shared decision making approach are healthcare professional-depending.

### Limits of the study and considerations for future studies

This study presents some points of weakness: the sample selection process and the may have led to selection biases. Therefore this pilot study cannot be considered as fully representative of the hospitalized population in Italy; moreover, observational studies are unable to supply strong evidence of causal connections among the different variables. Since these topics have been assessed with questionnaires, the interviewed subjects may have omitted some information about their real perception. On the other hand this pilot study gave the opportunity to focus on different aspects of shared decision making approach, to determine which dimensions of the patient-doctor relationship are connected to it and how does it affect organizational outcomes. This pilot study could represent the starting point to perform more specific multicentre studies with larger sample sizes.

## Conclusions

The pilot study outlined the main factors associated with a perceived shared decision making approach: interestingly, they appear to be all healthcare professional-related. Particularly, high quality and understandable information, try meeting patients needs and requests, spending a proper amount of time with them and taking into account their opinions on a larger scale, are all factors that this study found to be associated with a shared decision making approach.

The existing literature mainly focus upon patient-related aspects to understand SDM. This pilot study, other than confirming how these features are important in the decision-making processes, could also represent an opportunity to capture and deepen through other aspects of the shared decision-making construct. In particular it allows to rethink and reorganise some aspects regarding information about the therapeutic process, the amount of time to be spent on explaining the patients all the crucial aspects of their hospital stay, but also tailoring these actions on the basis of age and needs of the patients.

Thus, the restructure of decision-making from a *paternalistic* model to a *shared* one, in order to be effective, must take into account the change of some healthcare professional culture.

### Ethical standards

The authors assert that all procedures contributing to this work comply with the ethical standards of the relevant national and institutional committees on human experimentation and with the Helsinki Declaration of 1964 and its later amendments.

## Abbreviations

DRG: Diagnosis Related Group
SDM: Shared Decision Making
